# Visualization of SNPs with t-SNE

**DOI:** 10.1371/journal.pone.0056883

**Published:** 2013-02-15

**Authors:** Alexander Platzer

**Affiliations:** Gregor Mendel Institute, Vienna, Austria; Dana-Farber Cancer Institute, United States of America

## Abstract

**Background:**

Single Nucleotide Polymorphisms (SNPs) are one of the largest sources of new data in biology. In most papers, SNPs between individuals are visualized with Principal Component Analysis (PCA), an older method for this purpose.

**Principal Findings:**

We compare PCA, an aging method for this purpose, with a newer method, t-Distributed Stochastic Neighbor Embedding (t-SNE) for the visualization of large SNP datasets. We also propose a set of key figures for evaluating these visualizations; in all of these t-SNE performs better.

**Significance:**

To transform data PCA remains a reasonably good method, but for visualization it should be replaced by a method from the subfield of dimension reduction. To evaluate the performance of visualization, we propose key figures of cross-validation with machine learning methods, as well as indices of cluster validity.

## Introduction

SNPs are a major part of the extracted information from individual genomes. With the vast amount of NGS data, 100.000 s of SNPs can be found in a population today; naturally these are somewhat difficult to visualize. The most traditional method is PCA [1], which is still used in the majority of biology articles (e.g. [2]–[4]). PCA is designed for an orthogonal transformation, resulting in a number of components equal than or less to the number of original variables. These components are usually sorted for their explained variance.

At that point the assignment of a causing effect to the first components is attempted (e.g. [5]–[7]). For a correct assignment several constraints should be fulfilled [8].

Another usage is to plot the data with 2–3 higher components with primarily the first two or three principal components being plotted [2]–[4]. Due to the occasionally somewhat unsightly diagrams, several approaches to improve visualization with PCA have been developed (e.g. [9]).

In another field, that of machine learning, this problem of data reduction, often especially for visualization, has developed into its own subfield, ‘dimension reduction’, which was first outlined with the introduction of the term ‘the curse of dimensionality’ [10]. In this field several other methods have been developed since PCA, such as Sammon mapping [11], Isomap [12], Locally Linear Embedding [13], Classical multidimensional scaling [14], Laplacian Eigenmap [15], m-SNE [16], t-SNE [17], and others.

In this article we will focus on t-SNE as one of these newer methods and compare it with PCA in several ways.

The first step in comparing visualizing methods is of course to take several complex data sets, make diagrams, and discuss them. Decisions on aesthetic or artistic value may be made, but naturally more or less solid key figures for contrasting would be desirable.

The question of the quality of a visualization can be split in two parts: how well is the data structured; and how much (correct) insight can be obtained from it?

For biological data, the second question can often get out of hand; we will rather focus here on the first question.

Regarding the question of the structuredness of data, there exist long-known indices of cluster validity, such as Dunn's Validity Index [18], Silhouette Validation Method [19], and others (for an overview see [20]). But the property of structure can also be approached from another perspective: How easily may a model be built for the transformed data?

This question can be answered with splitting the data in two parts, use one part for constructing a model and the other to test it. The easier the structure of the data, the higher should be the validation key figure, assuming the model learning method makes an equal effort. We choose several machine learning methods for this purpose and compare their results for the different transformed data.

Here we show a comparison between the common PCA and the newer t-SNE on several large SNP datasets with a number of evaluating key figures.

## Results

**Figure 1:**
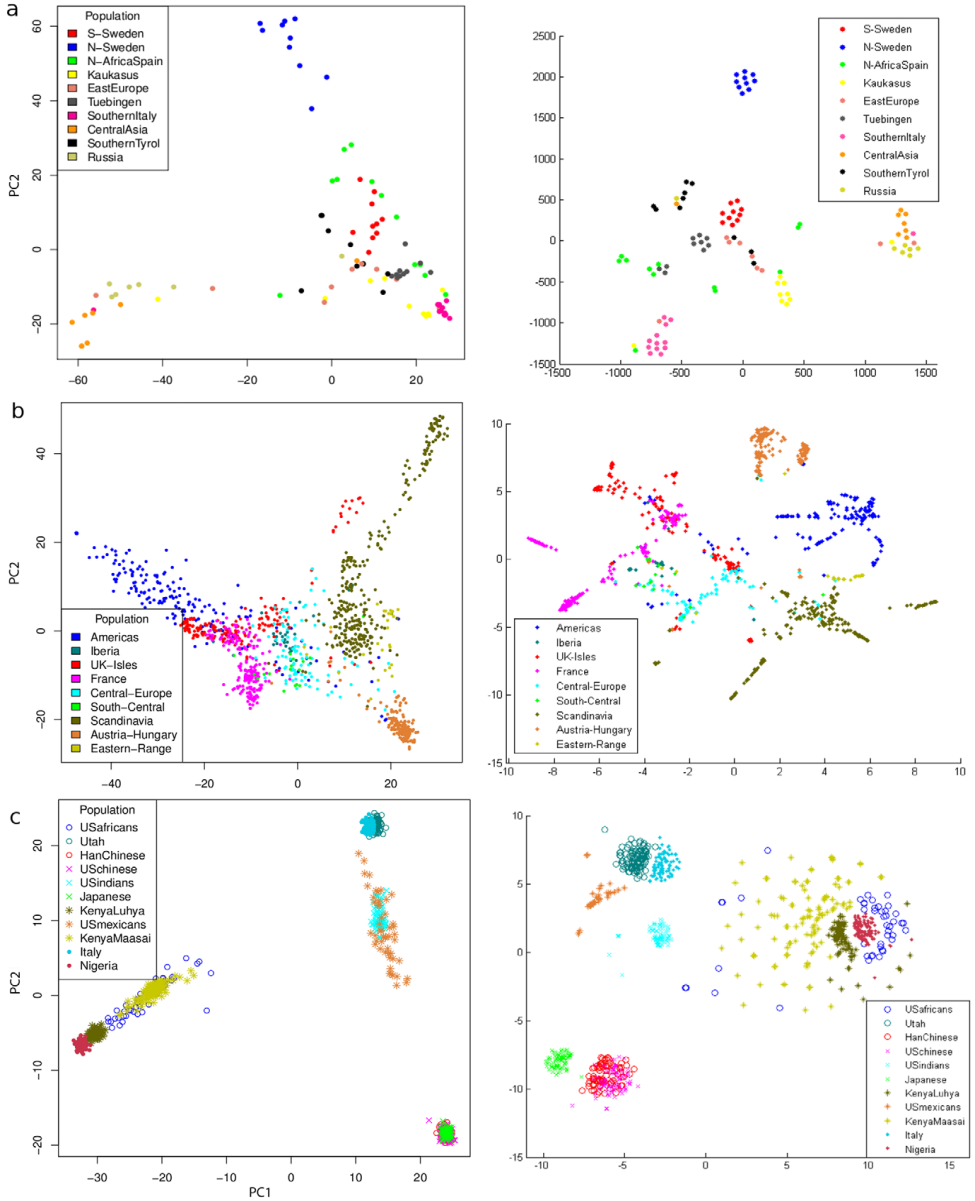
SNP data transformed with PCA and t-SNE 1/2. On the left is a PCA-plot with the first two components, on the right a t-SNE-plot of the very same data from each data source. Data sources: Panel (a) is from the 1001 genomes project, (b) from the RegMap panel and (c) from hapmap3 r2.

**Figure 2:**
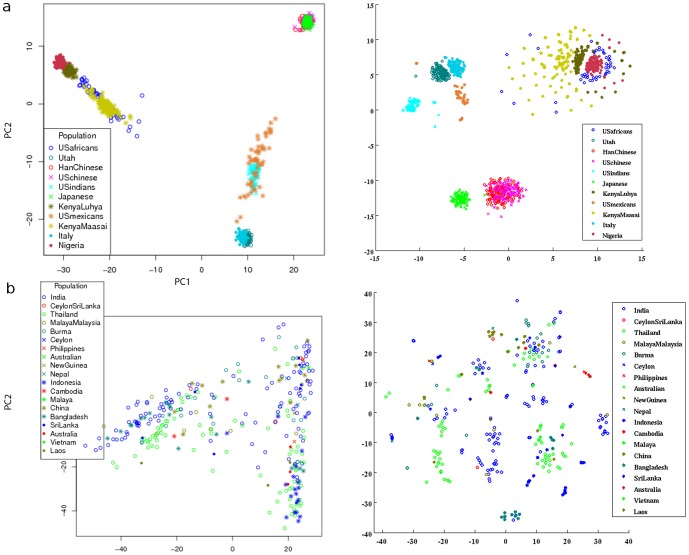
SNP data transformed with PCA and t-SNE 2/2. On the left is a PCA-plot with the first two components, on the right a t-SNE-plot of the very same data from each data source. Data sources: Panel (a) from hapmap3 r3 (compare with Fig. 1c) and (b) from the Rice Haplotype Map Project (only wild type where the label information was available).

In Figure 1 and Figure 2 we show the visual results of our chosen large SNP data sources transformed with PCA and with t-SNE. In light of the good separation, we should repeat here that both methods are unsupervised, that is, neither methods received labels and the colors were added after transformation. Visually, the t-SNE transformed data looks ‘nicer’ (our opinion and that of nearly all colleagues). The only mentioned drawback is that no extra biological information can be seen from the diagrams on the right. Here we will leave for discussion a final conclusion on the amount of biological impact and focus on the structuredness of the data. For this purpose, Table 1 contains the respective cluster validity indices for all diagrams, the values of Dunn's Validity Index, and the Silhouette Validation Method. The higher these values, the better the cluster separation, which corresponds to the structuredness. Both methods rely on the pairwise distances of the data points. Dunn's index can be used in different ways: We took the average function (the diameter of a cluster and the distance of clusters are defined generically at this method; for both we choose the average of pairwise distances). It is to mention that a Silhouette value lower than zero makes not much sense in terms of validation, as it means a random label assignment would be ‘better’. This occurs for the rice data (Figure 2b) because several clusters appear to consist of more than one real cluster, which are surrounding other clusters. Either the label ( = country) is a too low resolution or the location is not one of the largest effects in this data.

**Table 1:** Dunn's Validity Index and Silhouette Validation Method of the transformed SNP data.

Data	Dunn's Validity Index			Silhouette Validation Method		
	*PCA*	*t-SNE*	*Diff*	*PCA*	*t-SNE*	*Diff*
*1001 genomes*	0.52 (0.09)	0.61 (0.07)	0.09	0.07 (0.04)	0.22 (0.04)	0.15
*RegMap*	0.50 (0.06)	0.50 (0.04)	0.00	0.08 (0.02)	0.15 (0.02)	0.07
*Hapmap3R2*	0.16 (0.01)	0.25 (0.02)	0.09	0.27 (0.02)	0.31 (0.02)	0.04
*Hapmap3R3*	0.16 (0.01)	0.35 (0.01)	0.19	0.26 (0.02)	0.32 (0.02)	0.06
*Rice*	0.06 (0.07)	0.10 (0.10)	0.04	−0.54 (0.04)	−0.46 (0.04)	0.08

The values of two indices of cluster validity as a measure for structuredness of the different transformed data. As a comparison between PCA and t-SNE the diff(erence) column is expressive. The number in brackets is the standard deviation of the index with 1000 permutations of the labels.

As a more general measure of the structuredness of the transformed data, we formulate it as a supervised classification problem. The underlying rationale is that, if the data is well-structured, it should be easier for any method to construct a good model for it. We choose here C4.5, PART, Neural Networks, and naïve Bayes [21]–[24]. To judge a method's performance on a dataset we use the percent correctly classified of the 10-fold cross-validation. These results are presented in Table 2. Here, the difference in this key figure between PCA and t-SNE transformed data of the same source using the same learner should express the difference in structuredness of the two transformations. Mean values and standard deviations are only there per population as these populations are not fully comparable.

**Table 2:** Percent correctly classified with various machine learning methods acting on transformed SNP data.

1001 genomes project			RegMap		hapmap3 r2		hapmap3 r3		Rice	
%	*PCA*	*t-SNE*	*PCA*	*t-SNE*	*PCA*	*t-SNE*	*PCA*	*t-SNE*	*PCA*	*t-SNE*
*C4.5*	55.6	72.7	79.2	89.7	72.9	90.5	72.9	87.5	41.3	66.6
*PART*	60.6	76.8	77.6	89.1	72.7	90.9	73.3	87.6	39.7	64.9
*Perceptron*	67.7	76.8	80.7	85.8	70.3	85.1	72.2	84.8	50.5	56.4
*Naive Bayes*	62.6	75.8	75.2	80.3	74.6	87.2	71.8	84.1	40.7	42.3
*Mean diff.*	13.9		8.1		15.8		13.5		14.5	
*St.dev.*	3.6		3.4		2.6		1.2		12.5	

The percent correctly classified as a measure how easy a model can be learned. As comparison between PCA and t-SNE, the respectively difference between these two columns is expressive. All models are better than random.

For our large SNP data sources we selected the 1001 genomes project [25], the RegMap panel [26], hapmap3 release 2/3 [27] and the Rice Haplotype Map Project [28]. We picked a subset from the 1001 genomes project, firstly because it seemed at the time of analyzing that the data would not be fully complete in the near future, and secondly, for the equal class sizes. With very unequal class sizes, both PCA and t-SNE suffer, as we could see with different unequal subsampling (not shown), and as also stated by [29], [30]. *Class* is here and later referring to the labels of the data records, which are geographic locations in this paper. The next three datasets are taken as they were released, whereas the last dataset (Rice) was filtered for wild rice and for available labels ( = country).

The species of the first two datasets is *Arabidopsis thaliana*, the species of the next two is human (they are just different releases of the same effort). The last dataset is from a collection of rice. For all species a solid assumption seems to be that a large effect in the genomes is linked with their geographic location [31], [32].

As can be seen, all key figures to measure the structuredness of the transformed data point in the same direction (except for the RegMap data, where the Dunn Index is the same). A clear answer to the question of which transformation leads to better structured data thus materializes: there needs to be a movement away from PCA.

## Discussion

The main purpose of this paper is to show an approach for testing possible transformations of SNP/biological data to 2 dimensions for visualization. Many more methods exist than t-SNE and PCA [33], though some do seem theoretically and practically outperformed by others.

SNPs are one of the largest sources of new data in biology, but until now none of this data has been in main machine learning repositories (e.g. [34]). This will change in the future as certain SNP data generating projects finalize.

We made two attempts to measure structuredness, which strongly correlates to what most consider the better scatter plot. The sources here are, on the one hand, merely much discussion with no exhaustive survey. But our intention, on the other hand, was to express this in numbers from the start. Our first approach uses cluster validity key figures, despite their known weaknesses [35]. Our second approach uses machine learning methods, following the rationale: if a moderately complex algorithm can more easily gain some ‘understanding’, and/or build a relatively better internally validated model, possible human insight should correlate to that. As measure for the machine learning methods we use the percent correctly classified.

For machine learning methods themselves, there is of course only little gain, since other approaches [33] exist to deal with (too) many dimensions than to transform them to exactly two. Newer methods of this type are usually able to perform better with the full data, or with data not more than *sufficiently* reduced [36].

By performance we mean the result, not the computational effort, which can sometimes overload the frame. In the context of machine learning method performance, transformation of the data to two dimensions can be seen as a loss of information, which could be described by how much these methods lose in constructing models. The measure here could again be the percent correctly classified of the 10-fold cross-validation. The transformation that lets to the smallest decrease for all methods eligible for this classification problem should be judged better.

The four machine learning methods were not the only tested methods; we choose these four because of their high performance.

As mentioned above the structuredness of the transformed data is merely the first part of the various biological questions, the second always regarding the biological impact. There are several systematic attempts to directly translate it into biological information [2], [5]–[7]. Some appear quite convincing, while others seem more tweaks of the transformation. Nonetheless, with these attempts some insights have been obtained in this manner (e.g. [5]–[7]).

There may be other constraints that a dimension reduction method should fulfill to gain biological insight in other than standard classification problems. Like clustering in general this may simply remain ill-defined [37], [38].

## Materials and Methods

### PCA

For PCA, the build-in R function *prcomp()* is used.

### t-SNE

This method is presented in [17].

Beside the pseudocode of the simple version (Figure algorithm 1 in [17]), several ‘tricks’ and heuristics are used to make the results more attractive and/or the computation faster. All parameters for these ‘tricks’ are set within the method.

In the article ([17]), several other methods are compared and likely reasons for their worse performance are discussed. Some weaknesses also remain for t-SNE:

**Dimension reduction for more than 3 dimensions:** This was not a topic in designing t-SNE or in first testing, as it is irrelevant for visualization.

**Curse of intrinsic dimensionality:** Besides the general issue that dimension reduction always means that some information is lost, this targets the local linearity assumption of the method.

**Non-convexity of the t-SNE cost function:** This is one reason for the need for heuristics and tricks in the computation and the risk of not ending in the global optimum.

For t-SNE the matlab reference implementation is used [39].

There are two parameters for this implementation: *init_dims* and *perplexity*. *init_dims* is a preprocessing reduction with PCA to eliminate the most likely noise with skipping components with virtually no variance; it makes the computation faster. *perplexity* is used as defined in information theory, for example in [40]. Perplexity can be interpreted in this method as a smooth measure of the effective number of neighbors.

Unfortunately this version is restricted to 32bit, which entails a 2GB memory limit. There are other reference implementations, but all are restricted in memory usage at the moment.

Our chosen data sources would have required more total memory; to still allow the analysis, the data was downsampled to fit in 2GB memory. The same downsampled data was used also for the PCA.

### PCA vs. t-SNE

The most notable differences between the methods PCA [1] and t-SNE [17]:

- PCA splits the data into n components, sorted for variance (where n is the number of variables), whereas t-SNE squeezes all information in m components (where m is freely to choose, in case of plots m = 2)
- PCA is a static transformation: with one input there is always exactly one output (conditions for ambiguous cases are also precisely defined) t-SNE is a non-static transformation: with the same input there are different outputs possible, especially as the method is till now only feasible as more or less stepwise optimization; but also if the best value in terms of the cost function is always found, there will be several results because the method/optimization-criteria is rotation and scale-invariant
- PCA has several constraints [8], which are tackled in t-SNE
- PCA is an orthogonal linear transformation, whereas t-SNE is a nonlinear reduction, which ‘components’ are not constrained to be orthogonal.

The first of these points is the main convincing reason why PCA should not be the only plot in case of high dimensional data. As long as the number of dimensions is not too high, it is more likely that the first few (for a plot = 2 or 3) PCA components explain a lot of the data variance. If the first few components explain only little variance, then there is a big gain if a method integrates the rest of the data well, or put it in a different way: in a PCA plot there is always the information of n-2 components left out, where in t-SNE all information is tried to be combined.

Of course, if one of the largest effects in the data is perfectly correlating with the first two PCA components, then this transformation would be ‘better’ in terms of this effect. In SNP data this is usually not the case, otherwise a lot of published plots would look different and also the conclusion of this paper would be the opposite.

### Data sources

The 1001 genomes project [25] is one of the largest sources of SNP/genomic data for *Arabidopsis thaliana*, even though the data generation of this project is not finished. We used a subset of 99 individuals, selected for equal class sizes.

The Regional Mapping Project is another source for *Arabidopsis thaliana*. Though it has a lower resolution of SNPs, it is already finished. We have taken the same 1090 individuals as in the article's [26] PCA-plot.

The HapMap Project [27] is a large source of human genetic variation. We used the data from the second release of phase III, 988 individuals' sets of SNPs. We used also the third release of phase III as own dataset, because it was not sure at last if all issues were already resolved within (1198 individuals, should be a superset of the second release).

The Rice Haplotype Map Project [28] is the largest source of SNP/genomic data for rice. We have filtered here for the wild rice (species Oryza rufipogon) where the country of origin was available in the database (305 individuals).

### Indices of cluster validity

The transformed and labeled data can be seen as a result of a clustering method, although it is not gained in that manner: As result from clustering the labels would be assigned through the clustering method, whereas in our case the labels are the true (external) classes and the values of the variables are ‘generated’ ( = transformed original values). That means that the problem of judging the structuredness of our transformed data with the true classes is similar to judging the result of a clustering. For this reason we are able to use internal evaluation methods, although it is an external validation.

We choose Dunn's Validity Index [18] and the Silhouette Validation Method [19] for this purpose.

### Dunn's Validity Index and Silhouette Validation Method

A good short description of both methods can be found in Wikipedia ([41], [42]). Both methods rely on the pairwise distances of the data points within a cluster in comparison with distances within different clusters. Beside the chosen distance/dissimilarity, the main difference is that the Dunn Index looks for the worst combination (the maximal intra-cluster distance to the minimal inter-cluster distance), whereas the Silhouette Validation Method is taking the average of all cluster combinations (more precisely, the Silhouette Validation Method is originally defined for two clusters and we (/our chosen implementation) took the arithmetic mean of all combinations).

For Dunn's Validity Index we used the R package ‘clv’ [43] and for the Silhouette Validation Method we used the R package ‘cluster’ [44].

### Classification methods

For constructing models for classification we use four standard machine learning methods:

- The well-known tree learner C4.5 [21] and the not very widely used method PART [23] relying on C4.5.
- A Neural Network [22] with one hidden layer (5–7 hidden nodes).
- Naïve Bayes [24]

The analysis with these classification methods was performed with WEKA [45].

## References

[1] Pearson K (1901) On Lines and Planes of Closest Fit to Systems of Points in Space. Philosophical Magazine 2: 559–572.

[2] Sun Z, Chai HS, Wu Y, White WM, Donkena KV, et al (2011) Batch effect correction for genome-wide methylation data with Illumina Infinium platform. BMC Med Genomics 4: 84. 22171553 10.1186/1755-8794-4-84 PMC3265417

[3] Swingley WD, Meyer-Dombard DR, Shock EL, Alsop EB, Falenski HD, et al (2012) Coordinating environmental genomics and geochemistry reveals metabolic transitions in a hot spring ecosystem. PLoS One 7: e38108. 22675512 10.1371/journal.pone.0038108 PMC3367023

[4] Zhou H, Muehlbauer G, Steffenson B (2012) Population structure and linkage disequilibrium in elite barley breeding germplasm from the United States. J Zhejiang Univ Sci B 13: 438–451. 22661207 10.1631/jzus.B1200003 PMC3370289

[5] Hurtado MA, Racotta IS, Arcos F, Morales-Bojorquez E, Moal J, et al (2012) Seasonal variations of biochemical, pigment, fatty acid, and sterol compositions in female Crassostrea corteziensis oysters in relation to the reproductive cycle. Comp Biochem Physiol B Biochem Mol Biol 10.1016/j.cbpb.2012.05.011 22613818

[6] Jarzynska G, Falandysz J (2011) Selenium and 17 other largely essential and toxic metals in muscle and organ meats of Red Deer (Cervus elaphus)–consequences to human health. Environ Int 37: 882–888. 21429582 10.1016/j.envint.2011.02.017

[7] Yu Z, Tan BK, Dainty S, Mattey DL, Davies SJ (2012) Hypoalbuminaemia, systemic albumin leak and endothelial dysfunction in peritoneal dialysis patients. Nephrol Dial Transplant 10.1093/ndt/gfs075 PMC3520081 22516624

[8] A Tutorial on Principal Component Analysis. Available: http://www.snl.salk.edu/~shlens/pca.pdf. Accessed: 2013 Jan 21.

[9] Lu H, Plataniotis KN, Venetsanopoulos AN (2008) MPCA: Multilinear Principal Component Analysis of Tensor Objects. IEEE Trans Neural Netw 19: 18–39. 18269936 10.1109/TNN.2007.901277

[10] Bellman RE (1961) Adaptive Control Processes. Princeton, NJ: Princeton University Press.

[11] Sammon JW (1969) A Nonlinear Mapping for Data Structure Analysis. Ieee Transactions on Computers C 18: 401-&.

[12] Tenenbaum JB, de Silva V, Langford JC (2000) A global geometric framework for nonlinear dimensionality reduction. Science 290: 2319-+. 11125149 10.1126/science.290.5500.2319

[13] Roweis ST, Saul LK (2000) Nonlinear dimensionality reduction by locally linear embedding. Science 290: 2323–2326. 11125150 10.1126/science.290.5500.2323

[14] Torgerson WS (1952) Multidimensional Scaling: I. Theory and Method. Psychometrika 17: 401–419. 10.1007/BF02289530 5217606

[15] Belkin M, Niyogi P (2002) Laplacian eigenmaps and spectral techniques for embedding and clustering. Advances in Neural Information Processing Systems 14, Vols 1 and 2 14: 585–591.

[16] Xie B, Mu Y, Tao DC (2010) m-SNE: Multiview Stochastic Neighbor Embedding. Neural Information Processing: Theory and Algorithms, Pt I 6443: 338–346.

[17] van der Maaten L, Hinton G (2008) Visualizing Data using t-SNE. Journal of Machine Learning Research 9: 2579–2605.

[18] Dunn JC (1973) A Fuzzy Relative of the ISODATA Process and Its Use in Detecting Compact Well-Separated Clusters. Journal of Cybernetics 3: 32–57.

[19] Rousseeuw PJ (1987) Silhouettes - a Graphical Aid to the Interpretation and Validation of Cluster-Analysis. Journal of Computational and Applied Mathematics 20: 53–65.

[20] Halkidi M, Batistakis Y, Vazirgiannis M (2001) On clustering validation techniques. Journal of Intelligent Information Systems 17: 107–145.

[21] Quinlan JR (1993) Programs for Machine Learning: Morgan Kaufmann Publishers.

[22] Rumelhart DE, Geoffrey E Hinton, and R. J Williams (1986) Learning Internal Representations by Error Propagation. In: Parallel distributed processing: Explorations in the microstructure of cognition. Cambridge: MIT Press. pp. 318–362.

[23] Frank E, Witten I. Generating Accurate Rule Sets Without Global Optimization; 1998; Shavlik. Morgan Kaufmann Publishers, San Francisco, CA.

[24] Zhang H (2005) Exploring conditions for the optimality of Naive bayes. International Journal of Pattern Recognition and Artificial Intelligence 19: 183–198.

[25] Weigel D, Mott R (2009) The 1001 Genomes Project for Arabidopsis thaliana. Genome Biology 10. 10.1186/gb-2009-10-5-107 PMC2718507 19519932

[26] Horton MW, Hancock AM, Huang YS, Toomajian C, Atwell S, et al (2012) Genome-wide patterns of genetic variation in worldwide Arabidopsis thaliana accessions from the RegMap panel. Nature Genetics 44: 212–216. 22231484 10.1038/ng.1042 PMC3267885

[27] Altshuler DM, Gibbs RA, Peltonen L, Dermitzakis E, Schaffner SF, et al (2010) Integrating common and rare genetic variation in diverse human populations. Nature 467: 52–58. 20811451 10.1038/nature09298 PMC3173859

[28] Huang X, Kurata N, Wei X, Wang ZX, Wang A, et al (2012) A map of rice genome variation reveals the origin of cultivated rice. Nature 490: 497–501. 23034647 10.1038/nature11532 PMC7518720

[29] Khalilia M, Chakraborty S, Popescu M (2011) Predicting disease risks from highly imbalanced data using random forest. Bmc Medical Informatics and Decision Making 11. 10.1186/1472-6947-11-51 PMC3163175 21801360

[30] Lin WJ, Chen JJ (2012) Class-imbalanced classifiers for high-dimensional data. Brief Bioinform 10.1093/bib/bbs006 22408190

[31] Nothnagel M, Lu TT, Kayser M, Krawczak M (2010) Genomic and geographic distribution of SNP-defined runs of homozygosity in Europeans. Hum Mol Genet 19: 2927–2935. 20462934 10.1093/hmg/ddq198

[32] Sharbel TF, Haubold B, Mitchell-Olds T (2000) Genetic isolation by distance in Arabidopsis thaliana: biogeography and postglacial colonization of Europe. Mol Ecol 9: 2109–2118. 11123622 10.1046/j.1365-294x.2000.01122.x

[33] Laurens van der Maaten EP, Jaap van den Herik (2009) Dimensionality Reduction: A Comparative Review. Tilburg: Tilburg University.

[34] Asuncion AFaA (2010) UCI Machine Learning Repository. University of California, Irvine, School of Information and Computer Sciences.

[35] Christopher D. Manning PR, Hinrich Schütze (2009) An Introduction to Information Retrieval. Cambridge, England: Cambridge University Press.

[36] Amir Globerson NT (2003) Sufficient Dimensionality Reduction. Machine Learning Research 3: 1307–1331.

[37] Ackerman M, Ben-David S. Clusterability: A Theoretical Study; 2009; Clearwater Beach, Florida, USA.

[38] Pau G (2010) Clustering and classification with applications to microarrays and cellular phenotypes. Bressanone-Brixen, Italy: Computational Statistics for Genome Biology 2010.

[39] t-Distributed Stochastic Neighbor Embedding - Implementations. Available: http://homepage.tudelft.nl/19j49/t-SNE.html. Accessed: 21 June 2012.

[40] Brown PL, Della Pietra V, Lai JC, Mercer RL (1992) An estimate of an upper bound for the entropy of English. Computational Linguistics 18: 31–40.

[41] Dunn index – Wikipedia, The Free Encyclopedia. Available: http://en.wikipedia.org/w/index.php?title=Dunn_index&oldid=511861769. Accessed: 7 Jan 2013.

[42] Silhouette (clustering) – Wikipedia, The Free Encyclopedia. Available: http://en.wikipedia.org/w/index.php?title=Silhouette_(clustering)&oldid=528712368. Accessed: 2013 Jan 7.

[43] Tibshirani R, Walther G, Hastie T (2000) Estimating the number of clusters in a dataset via the Gap statistic. 63: 411–423.

[44] Struyf A, Hubert M, Rousseeuw P (1997) Clustering in an Object-Oriented Environment. Journal of Statistical Software 1: 1–30.

[45] Frank E, Hall M, Trigg L, Holmes G, Witten IH (2004) Data mining in bioinformatics using Weka. Bioinformatics 20: 2479–2481. 15073010 10.1093/bioinformatics/bth261

